# New York Odontological Society

**Published:** 1894-04

**Authors:** 

**Affiliations:** New York Odontological Society


### Reports of Society Meetings.

### NEW YORK ODONTOLOGICAL SOCIETY.

A REGULAR meeting of the New York Odontological Society was held on Tuesday evening, January 16, 1894, at the New York Academy of Medicine, No. 17 West Forty-third Street, New York City, with the President, Dr. Brockway, in the chair.

### INCIDENTS OF OFFICE PRACTICE.

*Dr. S. G. Perry.*—At the meeting held in Brooklyn a week ago last Monday, Dr. Darby read a paper on the subject of filling-materials, and took occasion to speak very favorably of tin-foil, and also to show before that society some specimens of tin which had been shaved off a revolving wheel of block tin attached to a lathe. He made the claim that pure tin, when prepared in that way with a fresh surface, possessed an adhesive property which is not found in tin-foil, and he showed some specimens of the tin prepared, and a large number of teeth which had been filled by his students, where the fillings had been built up to quite an extent,—as much as would sometimes be seen with gold, which of course would not be possible if the tin used had not a considerable adhesive property.

On Sunday last, in his laboratory, he exhibited to me his method of shaving off the tin, and I asked him to allow me to present some of the shavings to the members of this Society. I had my secretary put up fifty packages in this form, hoping there would be enough so that each one could try it. When it is first shaved off from the wheel there is no doubt it has cohesive properties which we have never found in tin-foil. Dr. Darby has used tin in this way for about eighteen years. I cannot tell you to what extent he has used it during that time, but he exhibited his old book of lectures to his students, in which he explained this as one of the ways in which tin could be used. I have tried it in quite a number of cases since he has shown it to me, and it certainly works well. How long that cohesive property will continue of course I do not know. I suppose it will be lost after being exposed to the air for a while.

It affords me an immense amount of satisfaction to see this ques tion brought up in this way. In a paper which I read in Brooklyn the month before, I spoke very strongly in favor of tin-foil. Tin was the material I learned first to fill teeth with, and I have never lost my love for it, not only because of the ease with which it can be worked, but because of its remarkable preservative properties. One of the greatest compliments that can be paid to tin-foil is that you have heard men all your life say, when a new kind of gold is introduced, "Why, it works more like tin-foil than any gold I ever saw."

To-day I saw the remnants of a tin filling on the approximal surface of a molar tooth which a patient of mine said I had put in about fifteen years ago. It had worn away until there was only a slight layer in the cavity. I had not seen her for a long time, and the tooth had remained for quite a long period only partially filled. I took the remaining portion of the filling out of the cavity, and put in a new one without touching the tooth with an instrument. I simply put in a new tin filling where the old one had been. I think it would be a great good fortune to patients as well as operators, if there could be a sort of revival of the use of tin-foil. I certainly feel that in our search for other materials we have overlooked this material. We have given it the go-by, and many of us without any reason. I believe that a revival of its use would be a good thing, used discriminately, of course, particularly in the grinding surfaces of young teeth, and on certain buccal surfaces, but not too much perhaps on proximal surfaces, because we all know there will occur that softening which is due probably to acid action; for a tin filling does better if the surface is exposed where there is a little attrition to keep it bright and smooth.

In years gone by I have many times filled young front teeth on the proximal surfaces, using tin-foil instead of gutta-percha. Of course it makes a little shadow between the teeth, but it does not discolor. Sometimes it will be found to be more durable than gutta-percha, where the teeth are not quite ready for gold fillings.

*Dr. Lord.*—I have recently made a change in the so-called sickle instrument, and I find it to be a very great improvement.

It consists simply in the grooving of the edges, which at once gives a thin and sharp edge in the place of a rectangular one.

The instrument should always be made the width that is desired, since to reduce the width would take out the groove. The thickness may be reduced, as will readily be seen, without disturbing the groove.

The sharpening may be done either with a fine sharp or new file or on a hone. The sharpening will of course, in time, more or less reduce the thickness. As it is intended that the groove shall be in both edges, the inner and the outer, the instrument can be used in the pushing quite as much as in the pulling effort, which is a most important feature.

It is well understood that the instruments for the removal of the tartar from between the teeth must be quite thin, but, as will be seen by the samples which I have brought to the meeting, the thin edge cannot receive much of a groove. The depth of the groove will depend upon or vary with the thickness of the blade of the instrument.

I have found, I may say, that instrument-makers do not readily get the right curve or the proper length of it; so I have had the instruments brought to me before they are tempered, that I might see that they were quite right.

I believe it will be found that these instruments are also most excellent for trimming the margins of foil fillings, as the edges so surely detect any roughness or want of perfect finish.

*Dr. Francis.*—I feel that the profession is greatly indebted to Dr. Lord for having introduced this instrument; also the original sickle-shaped instruments. I could hardly do without them in my practice, and have blessed Dr. Lord many times for devising them. After Dr. Perry had finished speaking about tin and Dr. Lord took the floor, I thought Dr. Lord would refer to a case which he spoke of on a former occasion, where a tin-foil filling had preserved a tooth in a lady's mouth for sixty years.

In my early practice tin-foil was much used, and even now I believe it is one of the best materials that can be employed for filling frail teeth. It is not used much at present for the reason, probably that it is much more difficult to introduce than plastic stoppings. A gentleman who has for many years lived in Europe called on me somewhat recently, showing me a tin-foil filling in a superior sixth-year molar which I put in twenty-five years before. He came the first time to have the tooth extracted, but I advised him to have it filled, and he consented. In the mean time other teeth on each side of it had disappeared, yet this one remained, and was in good condition. He called it his "memorial tooth."

An incident recently came to my observation which I think sufficiently interesting to relate. A few months ago a gentleman about thirty-five years of age called to have me extract the root of a tooth which he said was a source of much annoyance, causing severe headaches. On examination I found a partly decayed root of a left superior central incisor. This I removed. I said to him that it did not seem possible for this fragment of a root to cause such headaches, and requested him to call again in case he had further trouble. I met him a few weeks afterwards, when he said he would give me an incident of his early history which he thought might account for his headaches. When about fifteen years of age he had a decayed tooth (pointing to a space formerly occupied by a superior bicuspid) which he neglected to have filled. It decayed more and more until the crown broke entirely away. He was annoyed by collections of food crowding into the root, and was in the habit of using a pin to work out the collections. This practice was kept up for a long time, until he penetrated the canal its entire length. One day, while occupying his time in this manner, his attention for a time was diverted, and he forgot the pin. After a while it came to his mind, and he wondered what became of it. He did not recollect removing it, and yet he hardly believed it was still in the root, nor could he feel it with his nail. This occurred twenty years ago. Soon after I extracted the root of the incisor he experienced a peculiar sensation in the upper part of the nares which caused him to sneeze. Something rough passed out which he secured with his handkerchief. It proved to be a section of the pin, about one-third of it, with the head. A few days after another section appeared, which proved to be the pointed end. About one-third was still missing. He washed the parts secured, and kept them for me to examine. They were much eroded. The pin, it seems, during the twenty years had been gradually travelling from the bicuspid root up into the antrum and so into the nares, where it found its way out.

*Dr. Davenport.*—It has been my opinion for a number of years that many of our members were not interesting themselves sufficiently in the work of the Society, and the report of the executive committee, a portion of which has just been read, has very properly emphasized that idea. It stands to reason that in a society of this sort, composed as it is of fairly representative men, there are members who perform frequently peculiar operations which, if reported here, would help many of us in like cases of emergency, and if it did not give an idea of what to do, it might at least warn us, and show conclusively what not to do.

Every member of the Society ought to write a short casual communication describing some operation possessing peculiar, even if not original, features, and if we could have at every meeting one or two little descriptions of that sort I believe it would make our proceedings spicy, and would be a help to the membership as well as to our readers. If I may be allowed this evening to take the initiative, I should like to make a short report of an operation which to me was a little peculiar.

(For this report of Dr. Davenport, see page 253.)

*Dr. Watkins.*—Inasmuch as Dr. Davenport has taken us to task for not extending our ideas further than our own offices, I want to relate something which I think originated in my office many years ago, and which I do not think I ever told this Society, in regard to taking impressions, and occasionally in applying the rubber dam or perhaps the napkin in filling. Many people are very sensitive, and will become nauseated at the slightest touch at the back part of the tongue or the roof of the mouth. This can be overcome readily, I presume, in every case, by simply applying a little spirits of camphor to the tongue. A couple of drops will allay that sensation almost instantly, so you can take your impressions just as nicely as if the patient were not at all sensitive at any time.

In regard to tin-foil, I have used it considerably in connection with gold, nearly filling the cavity with tin-foil, and then simply covering with gold, using White's extra tough tin-foil, which seems to be quite cohesive, and three-quarters or perhaps seven-eighths filling the cavity, say large approximal cavities, and then covering the edge with gold, which will show when the patient laughs. I saw a case only a few days since which I filled in that way ten or eleven years ago. The gold was there and quite perfect, although the tin showed a little through the gold, so that it was not the bright natural color that gold should be.

*Dr. Rhein.*—I was very much interested in the remarks of Dr. Davenport with reference to uniting loose teeth by filling. Some of the members present may remember a case of this character that I presented in 1888, at the annual meeting of the First District Society. The reason that I advocated the method there employed, was to avoid as much as possible showing the gold. I have had considerable experience in holding teeth in this manner, having used the method that Dr. Davenport relates quite frequently. There arise many occasions where it becomes very valuable to be able to hold loose teeth together by uniting them by a wire filled into the cutting surfaces of the teeth, and this can be done almost without any unsightliness, granting that in some cases the work may require occasional repair. The case I reported at that time is in absolutely good condition to-day. That was done in 1887, and I have had to repair it slightly three times in different places. One point that I wish to bring out in the practical use of this method, which Dr. Davenport, I think, overlooked in doing his first operation, is this: he spoke of holding the wire with his fingers while inserting the filling. This seems scarcely adequate to bridge the proximal spaces with the filling, or to hold the wire and teeth sufficiently firm. The method I adopt is to embed all the teeth in oxyphosphate of zinc, having previously ligated them in proper position; make a mass thick enough to go around both the labial and lingual surfaces, if it is on the inferior jaw, so that the teeth are held thoroughly impacted in the mass. Allow that to harden thoroughly before the filling is commenced. You then have the teeth absolutely firm, and can do the entire operation, going along from one tooth to the other. If you picture it in your mind, you can readily imagine the oxphosphate passing between the approximal surfaces of the teeth so as to give you a bridge to build across, and afterwards trimming away the oxyphosphate. The point I wanted to especially bring out was this method of holding the teeth while the filling was being introduced. I do not believe the operation is a new one. I never claimed any originality for it *per se*, but I do say that I had never heard of its use in the treatment of pyorrhœa alveolaris before I introduced it. I have seen the revered Dr. Atkinson use the method in fractures of the maxillæ where the teeth had become loosened, and I suppose the idea obtained a foothold with me from witnessing his use of it for that particular form of work.

The secretary read a communication from Dr. H. G. Mirick, expressing his thanks to the Society for having proposed him as an honorary member.

Dr. J. Morgan Howe then presented his paper on "Alloys as Nostrums and as Subjects for Investigation."

(For Dr. Howe's paper, see page 211.)

*Dr. Crouse.*—I am not far enough along on the question of amalgams to talk about it. I never have found a manufacturer of amalgams who could make the same formula twice alike. I do not know of any (and I have made considerable tests) whereby they have gotten so they melted the metal and did not disturb some of the baser metal in the compound. I have not solved the question yet, although I hope to. I sent Dr. Howe some specimens a few days ago, and I believe he asked me for the formula. I dictated the formula, but I do not know whether I sent it to him. When the question of aluminum amalgam came out, I had some friends who were anxious to have me test it and see what I thought of it in a practical way. I went at it with a great deal of doubt, and did not believe in it, and took hold of it with the expectation of condemning the aluminum in it as an ingredient, but to my surprise and not to my dissatisfaction, I found that there seemed to be something in aluminum amalgam that I had not supposed would be there. Then I wrote to inquire about it of Carroll, to find out how much aluminum was in it, and he wrote me it was nearly all aluminum. I ordered ten ounces of his amalgam, and put it into three different hands, and not one of them found more than two per cent. of aluminum in it. Of course I was not much surprised at that, but I am surprised to know the effect of the aluminum. I took the same formula and added a little bit of gold to make it set a little quicker. I have made several experiments. The most important thing is to know what metals you want in amalgams, and the next thing is how to put those metals together and know that you have the proportions there. My superintendent was prejudiced in favor of fine amalgam, and I think you will notice it is finer than we had before. There should be a machine that would cut it nearly uniform in whatever grade you want it. I think a milling machine would give you an almost uniform grain.

This is a question I am very glad the Society is agitating. I am having a furnace made to see if we cannot get it without burning some of the baser metals out. At present it is not possible to put together two per cent. of aluminum and a certain amount of gold and copper and zinc, and know that they are all there when we get the compound out. I have also thought that the question of how amalgam will appear in different mouths is not settled.

*Dr. B. C. Nash.*—As I understand it, Dr. Howe experimented with several alloys as they came to him, and by a process of sifting obtained a number of batches, the grain of which varied in degree of fineness. In conversation with a manufacturer, I learned that the quality of grain in high-grade alloys is invariably fine, no matter if the file used in cutting it be coarse or fine.

*Dr. Hammond.*—A metallurgist, Dr. Crouse, has spoken of not being able to secure absolute uniformity in his alloys. That is possible. It is so possible, that by weighing your materials before putting them into the furnace and weighing the resulting ingots (taking, for instance, a forty-ounce batch), the loss may be from one to ten grains. Anything more than this is carelessness or inexperience in the experiment. I have had a great deal of experience in smelting different metals, and feel competent to say this. That there *is* a variation in the working many times, is true; it is not necessary, however, that it should be from a difference in the formula or accidental. The mere fact of cooling slower or faster will make a great deal of difference in the hardness of the amalgamation when it is worked up with mercury. If you chill it instantaneously, the amalgamation is almost impossible. I am speaking now of high grade alloys, which have a considerable proportion of silver, whereas, taking these same alloys and cooling them slowly, they work very much smoother.

Adjourned.

John I. Hart, D.D.S.,
*Editor New York Odontological Society.*

